# Supplementary material for: Patterns of Intron Gain and Loss in Fungi
Source: PLoS Biol. 2004 Nov 30;2(12):e422. doi: 10.1371/journal.pbio.0020422 (PMC532390; doi:10.1371/journal.pbio.0020422)
Supplement: Table S1 — Also available at http://genes.mit.edu/NielsenEtAl/. (4.3 MB ZIP). [file pbio.0020422.st001.zip › NielsenEtAl/html/216.html]

AN5629.1.NCU04044.1.MG00647.1.FG09250.1


```
 CLUSTAL W (1.82) Multiple Sequence Alignments - Introns Inserted


Sequence 1: MG00647.1	497 aa
Sequence 2: FG09250.1	486 aa
Sequence 3: NCU04044.1	493 aa
Sequence 4: AN5629.1	497 aa
Alignment Length: 498 aa
Number Identitical Residues: 370 aa
Alignment Score (without introns) 16222


MG00647.1 	MLSRTTATPLARVAARPACSVATQQQPARSFATVQDGTPAPKRTYGGLRDQDRIFQNLYG
NCU04044.1	MLSRTAAP----TKASARTLSRAAAEQCRTFATVQDGSANPVRHYGGLKDQDRIFQNLYG
FG09250.1 	MLSTRTA---------PKKVVGLSRTAVRGLATVQDG--APKRTYGGLRDQDRIFQNLYN
AN5629.1  	MISRAAAPSSTPLASLSSRSLRLQAPAARSFATVSDN-APPVHHHGGLKDQDRIFTNLYG
          	*:*  :*.. :   : .           * :***.*. . * : :***:****** ***.

MG00647.1 	RYPPDLKHAKKMGDWHKTKEIILKGHDW~IIGEIKASGLRGRGGAGFPSGLKW0SFMNFK
NCU04044.1	RYPPDLKHAKKMGDWHKTKEILLKGHDW~IIGEVKASGLRGRGGAGFPSGLKW0SFMNFK
FG09250.1 	RYPVDLKSARKMGDWHKTKEIILKGHDW~IISEVKASGLRGRGGAGFPSGLKW0SFMNFK
AN5629.1  	HHGADLKSAMKYGDWYRTKDIVLKGHDW0LISELKASGLRGRGGAGFPSGLKY0SFMNFK
          	::  *** * * ***::**:*:****** :*.*:******************: ******

MG00647.1 	DWDKDTKPRYLVVNADEGEPGTCKDREIMRKDPHKLIEGCLVAGRAMNATAAYIYIRGEF
NCU04044.1	DWDKDDKPRYLVVNADEGEPGTCKDREIMRKDPHKLVEGCLVAGRAMNATAAYIYIRGEF
FG09250.1 	DWDKDNKPRYLVINADEGEPGTCKDREIMRKDPHKLIEGCLISGRAMNATAAYIYIRGEF
AN5629.1  	DWDKDPRPRYLVVNADEGEPGTCKDREIMRKDPQKLIEGCLVVGRAMNANAAYIYIRGEF
          	***** :*****:********************:**:****: ******.**********

MG00647.1 	YHEAAVLQGAINEAYADGLIGKNACGSGYDFDVFVHRGGGAYVCGEETSLIESLEGKAGK
NCU04044.1	IQEAAILQNAINEAYADGLIGKNACGSGYDFDVYLHRGAGAYVCGEETSLIESLEGKPGK
FG09250.1 	VEEAAVLQRAINEAYAAGLIGKNACESGYDFDVYLHRGAGAYVCGEETSLIESLEGKPGK
AN5629.1  	YHEATVLQQAINEAYQAGLIGKNACGTGYDFDVFIHRGMGAYVCGEETSLIESLEGKAGK
          	 .**::** ******  ******** :******::*** ******************.**

MG00647.1 	PRLKPPFPAAVGLFGCPSTVANVETIAVAPTICRRGGSWFAGFGRERNQGTKLFCISGQV
NCU04044.1	PRLKPPFPAAVGLFGCPSTVANVETVAVAPTICRRGGNWFAGFGRERNQGTKLFCISGHV
FG09250.1 	PRLKPPFPAAVGLFGCPSTVTNVETVAVAPTICRRGGSWFAGFGRERNQGTKLFCISGHV
AN5629.1  	PRLKPPFPAAVGLFGCPSTVTNVETVAVTPTIMRRGASWFAGFGRERNAGTKLFCISGHV
          	********************:****:**:*** ***..********** *********:*

MG00647.1 	NNPCTVEEEMSIPMRELIEKHCGGVRGGWDNLLAVIPGGSSTPILPKNICDDQLMDFDAL
NCU04044.1	NNPCTVEEEMSIPMRELIDKHCGGVRGGWDNLLAVIPGGSSTPILPKHICDTQLMDFDAL
FG09250.1 	NNPCTVEEEMSIPMRELIDKHCGGVRGGWDNLLAVIPGGSSTPVLPKSVCDDQLMDFDAL
AN5629.1  	NNPCTVEEEMSIPLRELIDRHCGGVRGGWDNLKAVIPGGSSTPIIPKSVCDDQLMDFDAL
          	*************:****::************ **********::** :** ********

MG00647.1 	KDSQSGLGTAAVIVMDKSADVVRAIARLSHFYRHESCGQCTPCREGSKWTEQIMHRFEHG
NCU04044.1	KDSQSGLGTAALIVMDKSTDVVRAISRLSHFYRHESCGQCTPCREGSKWTEQIMKRFEKG
FG09250.1 	KDSQSGLGTAAVIVMDKSTDIVRAISRLSHFYRHESCGQCTPCREGSKWTEQMMKRFEKG
AN5629.1  	KDSQTGLGTAAVIVMDKSTDVVRAISRLSTFYKHESCGQCTPCREGSKWTLQMMQRLEKG
          	****:******:******:*:****:*** **:***************** *:*:*:*:*

MG00647.1 	QGRVREIDMLQELTKQVEGHTICA1LGEAFAWPIQGLIRHFRPELEKRMADFAEKTGGE-
NCU04044.1	QGREREIDMLQELTKQVEGHTICA1LGEAFAWPIQGLIRHFRPELEARIRKFAQENGGE-
FG09250.1 	QAREREIDMLQELTKQVEGHTICA1LGEAFAWPIQGLIRHFRPELEARMQKFAQENGGE-
AN5629.1  	QAREREIDMLQELTKQVEGHTICA~LGEAFAWPIQGLIRHFRPELEARIKEYSEGLGGQQ
          	*.* ******************** ********************* *: .:::  **:.

MG00647.1 	ALAGGWTHDTRQQGKLVAPGQ
NCU04044.1	ALAGGWQRNARQQGKLVSPGM
FG09250.1 	ALAGGWNHNAKSQGKLVSPGQ
AN5629.1  	PLAGGWHPNSRAEGKLISPGM
          	.*****  ::: :***::**
```
